# Undiagnosed hypertension and its determinants among hypertensive patients in rural districts of northwest Ethiopia: a mediation analysis

**DOI:** 10.1186/s12913-023-09212-1

**Published:** 2023-03-07

**Authors:** Destaw Fetene Teshome, Shitaye Alemu Balcha, Tadesse Awoke Ayele, Asmamaw Atnafu, Kassahun Alemu Gelaye

**Affiliations:** 1 Department of Epidemiology and Biostatistics, Institute of Public Health, College of Medicine and Health Sciences, University of Gondar, Gondar, Ethiopia; 2 Department of Internal Medicine, School of Medicine, College of Medicine and Health Sciences, University of Gondar, Gondar, Ethiopia; 3 Department of Health Systems and Policy, Institute of Public Health, College of Medicine and Health Sciences, University of Gondar, Gondar, Ethiopia

**Keywords:** Undiagnosed hypertension, Proportion, Mediator, Ethiopia

## Abstract

**Background:**

Early detection of hypertension is associated with improved blood pressure control and a reduced risk of cardiovascular diseases. However, in rural areas of Ethiopia, evidence is scarce where access to healthcare services is low. This study aimed to estimate the proportion of undiagnosed hypertension and identify its determinants and mediators among patients with hypertension in rural northwest Ethiopia.

**Methods:**

A community-based cross-sectional study was conducted from September to November 2020. A three-stage sampling technique was used to select a total of 2436 study participants. Blood pressure was measured using an aneroid sphygmomanometer two times, 30 min apart. A validated tool was used to assess participants’ beliefs and knowledge of hypertension. The proportion, determinants, and mediators of undiagnosed hypertension were determined among patients with hypertension. The regression-based approach used to calculate the direct and indirect effects of determinants of undiagnosed hypertension. Joint significance testing was used to determine the significance of the indirect effect.

**Results:**

The proportion of undiagnosed hypertension was 84.0% (95% CI: 81.4–86.7%). Participants aged 25–34 years (AOR = 6.03; 95% CI: 2.11, 17.29), who drank alcohol (AOR = 2.40; 95% CI: 1.37, 4.20), were overweight (AOR = 0.41; 95% CI: 0.18, 0.98), had a family history of hypertension (AOR = 0.32; 95% CI: 0.20, 0.53), and had comorbidities (AOR = 0.28; 95% CI: 0.15, 0.54) were significantly associated with undiagnosed hypertension. The mediation analysis revealed that hypertension health information mediated 64.1% and 68.2% of the effect of family history of hypertension and comorbidities on undiagnosed hypertension, respectively. Perceived susceptibility to hypertensive disease mediated 33.3% of the total effect of age on undiagnosed hypertension. Health facility visits also mediated the effect of alcohol drinking (14.2%) and comorbidities (12.3%) on undiagnosed hypertension.

**Conclusion:**

A higher proportion of hypertensive patients remain undiagnosed. Being young, drinking alcohol, being overweight, having a family history of hypertension, and having comorbidities were significant factors. Hypertension health information, knowledge of hypertensive symptoms, and perceived susceptibility to hypertension were identified as important mediators. Public health interventions aimed at providing adequate hypertension health information, particularly to young adults and drinkers, could improve knowledge and perceived susceptibility to hypertensive disease and reduce the burden of undiagnosed hypertension.

**Supplementary Information:**

The online version contains supplementary material available at 10.1186/s12913-023-09212-1.

## Background

Noncommunicable diseases, a major public health problem in Ethiopia [1, 2], account for about 37.5% of the disease burden and 43.5% of all deaths [1]. Hypertension is a major risk factor for cardiovascular diseases (CVD) deaths, which are the leading cause of death from non-communicable diseases in the country [3]. Hypertension affects more than 20% of adults in the country [4–6], making it a major public health concern. The pooled prevalence of hypertension in rural settings of the country was 18.5% [7]. In some rural areas of the country, the prevalence of hypertension can reach up to 25.3% [4].

In Ethiopia, only one out of every 67 hypertensive adults has their blood pressure (BP) under control, making it a difficult task [3]. Hypertension complications are now the leading cause of hospitalization and death in the country. A hospital-based study found that hypertension complications account for 11.3% of all medical admissions and 14.6% of all medical ward deaths [8]. A similar study at Mekelle Hospital found that hypertension was the cause of 66.2% of all stroke admissions [9].

Early detection of hypertension is a critical first step in the hypertension care cascade toward improving hypertension care [10], preventing the disease’s domino effect [11], and saving lives [12]. Individuals with hypertension diagnosed early will benefit from a healthier lifestyle, better treatment, effective BP control [13], and a lower risk of coronary heart disease and stroke [14]. Despite the benefits of early hypertension diagnosis and treatment, only a small proportion of hypertensive patients are aware of, treated for, and controlled for it, making hypertension an iceberg disease. A study conducted in high, medium, and low-income countries, for example, found that only 46.5% of those with hypertension were aware of their condition [15]. In a population-based cross-sectional study of 44 lower and middle-income countries, 39.2% of those with hypertension were aware of their hypertension [16]. A systematic review and meta-analysis of hypertension awareness in sub-Saharan Africa found that only 27% were aware of their disease status [17].

Hypertension prevention and management were not given attention in Ethiopian rural communities, where the majority (80%) of the population lived [18], with limited access to healthcare and a shortage of healthcare providers [19]. In Ethiopia, few studies and reports revealed that less than 40% of hypertensive patients were diagnosed, one-third of those diagnosed received treatment, and only 26% of those on treatment had adequate BP control [1, 4, 20]. The 2015 Ethiopian STEPS survey report also showed that only 2.8% of hypertensive patients received treatment [21]. The portion of the iceberg below the water line represented the community’s latent or undiagnosed cases of hypertension [22].

Reliable evidence on the prevalence and determinants of undiagnosed hypertension are essential for developing effective health policies and hypertension management strategies. Exploring the mechanism of effects and quantifying the impact of modifiable risk factors in causal pathways between the determinants and undiagnosed hypertension may help in the development of targeted, population specific public health interventions to reduce the community’s burden of undiagnosed hypertension. Hence, this study aimed to determine the proportion of undiagnosed hypertension and to identify determinants and mediating variables using mediation analysis in adult populations in rural districts of northwest Ethiopia.

## Methods

### Study design and setting

A community-based cross-sectional study was conducted in the Dabat and Gondar Zuria districts of Amhara National Regional State in northwest Ethiopia between September and November 2020. The study settings have been described in detail elsewhere [23].

### Study participants and sampling

Adult population aged 25 years and more who lived for at least 6 months in the study settings participated in the study. Pregnant women were excluded from the study because of the effect of pregnancy-induced hypertension. A single population proportion formula was used to estimate the 2436 adult population using a 13.25% estimated prevalence of undiagnosed hypertension [24], a 95% confidence level (α = 5%), a 2% margin of error, a design effect of 2, and a non-response rate of 10%. A three-stage sampling technique was used to select study participants. First, total of 20 kebeles (10 from each district) were selected using a simple random sampling technique. Then, villages from each kebele were selected using a simple random sampling technique. Finally, from each village, one participant per household was included using the lottery method.

### Data collection tools and procedures

A structured questionnaire adapted from the WHO STEP-wise approach to surveillance of non-communicable diseases [25] was used to collect the data on socio-demographics, behavioral, psychosocial stress levels, and clinical-related factors. The Ethiopian demographic health survey tool was used to assess wealth status of the participants. Existing litratures were used to collect data on hypertension health information, hypertension knowledge, health beliefs, healthcare access, travel time and distance to a nearby health facility, health insurance coverage, health seeking behavior and healthcare utilization, personal history of hypertension, and linkage to hypertension care and treatment. Height, weight, and BP were also measured. The health extension workers, data collectors (MSc in emergency and critical care nursing and public health officers with a master of public health degree), and supervisors received two days of theoretical and practical training on the disease condition. The practical session included the selection of the correct cuff size, proper body positioning during measurement, and conducting the BP measurement. Face-to-face interviews were performed using an Amharic version of the questionnaire. The research team closely monitored the data collection process daily.

### Measurement and operational definition

The rural community’s household assets were used to calculate the family’s wealth [26]. These were combined into a single wealth index and then divided into three equal-sized groups of poor, medium, and rich. Health insurance status is defined by the questions, “Is your family a member of a community-based health insurance?” Those who answered “no” were categorized as “uninsured” [27]. Those who answered “yes” were asked the question, “Does the insurance cover all health care costs?” Participants who answered “yes” to this question were classified as “fully or adequately insured,“ while those who answered “no” were classified as “underinsured.“ Alcohol consumption was assessed by asking participants if they had consumed alcohol in the previous 12 months and categorizing them as alcohol users if they had used alcohol either regularly or occasionally. Participants’ physical activity was assessed using three specific types of activities: walking, moderate-intensity activities, and vigorous-intensity activities in the past 7 days. Physical activity was classified as low if it was < 600 metabolic equivalent tasks (MET) min/week, moderate when it was between 600 and 2999 MET min/week, and high when it was ≥3000 MET min/week [28]. Self-reported comorbidity data were collected using yes/no questions such as “Have you ever been told by a health care provider that you have: cardiovascular disease (heart attack, stroke), diabetes mellitus, and chronic respiratory disease such as asthma, chronic kidney disease, or cancer?“ Participants with chronic comorbidities were defined as having at least one chronic comorbid condition [29].

A 24-items questionnaire was used to assess participants’ knowledge of hypertensive disease. It consisted of questions with yes (1), no (2), or don’t know responses (3). Negative questions were encoded in the opposite direction to the other items. Correct responses received a score of one, while incorrect or don’t know responses received a score of zero. The sum score for each subdomain was calculated, converted to percentage, and classified as having poor knowledge (< 50%), medium knowledge (50–75%), or good knowledge (> 75%) [30, 31]. The hypertension belief assessment tool (HBAT) was used to assess participants’ health beliefs about hypertension. The hypertension belief assessment tool has 23 items, including perceived susceptibility, perceived health-related severity, perceived socio-economic related severity, perceived benefits, perceived barriers, and self-efficacy. The items were measured on a five-point Likert scale, ranging from strongly disagree (1) to strongly agree (5), and were found to have good reliability and validity [32]. The mean score for each subdomain was computed by adding the scores of all items in each subdomain, divided by the total number of items in each subdomain. Participants were categorized as having high perceived susceptibility, perceived health-related severity, perceived socio-economic related severity, perceived benefits, perceived barriers, and self-efficacy if they scored mean and above the mean score, and low belief if they scored below the mean.

Blood pressure was measured in the sitting position on the left arm. A calibrated aneroid sphygmomanometers and stethoscopes were used to measure BP. The participants were asked whether they smoked, drank caffeinated beverages, and had been working within 30 min or not before the BP measurements [33]. Health extension workers measured the first BP and recorded it to the nearest 2 mmHg. The trained data collectors also took two BP measurements for each individual 30 min apart, one before and the other at the end of the interview. The average of the last two BP measurements was used to estimate the mean BP. Hypertension is defined as a mean systolic BP of ≥130 mmHg or a diastolic BP of ≥80 mmHg or regular use of antihypertensive treatment [34]. Undiagnosed hypertension is defined as individuals who responded that they had not been told by a healthcare provider that they had hypertension but who would be diagnosed with hypertension based on the average of BP ≥ 130/80 mmHg cut point. The weight of the participants was measured using a digital scale to the nearest 0.1 kg while they wore light clothing and without shoes. Participants’ heights were measured with a tape to the nearest 0.1 cm. Participants were asked to stand upright and straight, without shoes, with their heels together and their eyes forward. Body mass index was calculated by dividing one’s weight in kilograms by one’s height in meters squared (weight/height in m^2^), and classified as underweight (< 18.5), normal (18.5–24.9), overweight (25-29.9), and obese (≥ 30) [35].

### Data analysis

The data were entered into Epi Data 4.6 and analyzed with STATA 16. The data were cleaned, coded, and recoded. Frequency, percentages, and median with an interquartile range were used to describe the data. Texts, tables, and graphs were used to present the findings.

Mediation analysis was performed among hypertensive patients to explore the causal pathway from the independent variables to the dependent variable (undiagnosed hypertension) via mediating variables. The regression-based approach [36], consisting of three separate regression models, was used to estimate the path or regression coefficients. Regression Eq. 1 uses multivariable binary logistic regression to determine the determinants of undiagnosed hypertension (path c). Regression Eq. 2 uses binary logistic regression (binary mediators) and ordinal logistic regression (ordinal mediator variable) to examine the relationship between independent variables and potential mediators (path a) adjusted for covariates. Regression Eq. 3 employs a binary logistic regression model to predict undiagnosed hypertension from both mediators and independent variables (paths b and c’), while controlling for covariates. The results were presented as adjusted odds ratios (AORs) with 95% confidence intervals (CIs) and regression coefficient values.

The direct and indirect effects of each independent variables were calculated. The indirect effect is the portion of the total effect that can be explained by the mediator/s (path “a” and “b”). The indirect effect was estimated using the product method (ab) [37, 38] and the joint significance testing method was used to determine its significance at a p-value of 0.05. According to this simple rule of thumb, we infer support for mediation if two conditions are met: path “a” is statistically significant, and path “b” is also statistically significant [39]. The proportion of effect explained by each mediator was calculated by dividing the specific indirect effect of each mediator by the total effect, where the total effect was calculated as the sum of the total indirect effect (all mediators combined) and the direct effect and multiplied by 100% [40].

## Results

### Sociodemographic characteristics of participants

A total of 2423 study participants included in the study (response rate of 99.5%). The median age of the participants was 45 (IQR: 34–55) years. Of the participants, 1337 (55.2%) were female, 2396 (98.9%) followed Orthodox Tewahido Christianity by religion, 1989 (82.1%) were married, and 1698 (70.1%) could not read and write (Table 1).

**Table 1:** Sociodemographic characteristics of participants in rural areas of northwest Ethiopia, September-November 2020

Variables	Total participants (n = 2423)	Hypertension (n = 758)
Sex		
Male	1,086 (44.8)	304 (40.1)
Female	1,337 (55.2)	454 (59.9)
Age		
25–34	613 (25.3)	98 (12.9)
35–44	597 (24.6)	152 (20.1)
45–54	523 (21.6)	164 (21.6)
55–64	337 (13.9)	141 (18.6)
≥65	353 (14.6)	203 (26.8)
Religion		
Orthodox	2,396 (98.9)	751 (99.1)
Muslim	27 (1.1)	7 (0.9)
Marital status		
Single	147 (6.1)	25 (3.3)
Married	1989 (82.1)	582 (76.8)
Divorced	98 (4.0)	45 (5.9)
Widowed	189 (7.8)	106 (14.0)
Educational status		
Unable to read and write	1698 (70.1)	546 (72.0)
Able to read and write	429 (17.7)	138 (18.2)
Primary school or more completed	296 (12.2)	74 (9.8)
Occupational status		
Farmer	2324 (95.9)	739 (97.5)
Student	49 (2.0)	9 (1.2)
Others*	50 (2.1)	10 (1.4)
Wealth index**		
Poor	808 (33.4)	293 (38.7)
Medium	808 (33.4)	247 (32.6)
Rich	807 (33.3)	218 (28.8)

*Daily laborer, Merchant

**Income is categorized based on percentiles

### Behavioral and lifestyle characteristics of the participants

Among the participants, only 18 (0.8%) and 8 (0.3%) had ever chewed chat and smoked cigarettes, respectively. Most of the participants, 2293 (94.6%) had ever used alcohol; and 2208 (91.1%) used alcohol during the previous 12 months. Three hundred thirteen (12.9%) of the study participants had a low level of physical activity, 323 (13.3%) slept for less than six hours per day, and 257 (10.6%) had a high level of stress. Of the participants, 654 (27.0%) were underweight and only 78 (3.2%) were overweight (Table 2).

**Table 2:** Behavioral and lifestyle characteristics of study participants in rural areas of northwest Ethiopia, September-November 2020

Variables	Frequency	Percent
Ever smoked cigarette		
Yes	8	0.3
No	2445	99.7
Ever chewed chat		
Yes	18	0.8
No	2405	99.2
Ever used alcohol		
Yes	2293	94.6
No	130	5.4
Alcohol drinking within the last 12 months		
Yes	2208	91.1
No	215	8.9
Number of alcohol drinking days (n = 2208)		
Daily	121	5.5
5–6 days per week	151	6.8
3–4 days per week	249	11.3
1–2 days per week	732	33.2
1–3 days per month	671	30.4
Less than once a month	284	12.9
Level of physical activity		
Low	313	12.9
Moderate	583	24.1
High	1527	63.0
Sleep duration, hours		
< 6	323	13.3
≥6	2100	86.7
Stressful life		
Not at all	1359	56.1
Some extent	807	33.3
Much	257	10.6
Body Mass Index		
Underweight	654	27.0
Normal	1691	69.8
Overweight	78	3.2

### Clinical characteristics of the participants

Of the total participants, 275 (11.4%) had a family history of hypertension (FHH), 88 (3.6%) had CVD, 40 (1.7%) had chronic kidney disease, 40 (1.7%) had chronic obstructive respiratory diseases, and 36 (1.5%) had diabetes mellitus. One hundred sixty (6.6%) of the participants had either or all of the aforementioned comorbidities. Of hypertensive patients, 129 (17.0%) had a FHH, 34 (4.5%) had CVD, 17 (2.3%) had chronic kidney disease, 12 (1.6%) had chronic obstructive respiratory diseases, 2 (0.3%) had diabetes mellitus, and 59 (7.8%) had one or more of the aforementioned comorbidities.

### Health information, knowledge, and beliefs about hypertension

Of the total participants, 518 (21.4%) had heard any health information about hypertension. Of those, 388(74.9%), 166 (32.0%), 141 (27.2%), and 100 (19.3%) heard the health information from health workers, family, relatives, and mass media, respectively. Similarly, 185 (24.4%) of hypertensive patients heard any hypertension-related health information. Health workers, family, relatives, and the media were the sources of health information for 154 (83.2%), 56 (30.3%), 52 (28.1%), and 23 (12.4%) of hypertensive patients. Of the total participants, 496 (20.5%) had overall good knowledge of hypertension, 640 (26.4%) had good knowledge of common hypertensive symptoms, 1225 (50.6%) had good knowledge of lifestyle risk factors and preventive measures. Of the total participants, 957 (39.5%) had a high perceived susceptibility to hypertensive disease, 1394 (57.5%) had high perceived health-related severity of hypertension, 1248 (51.5%) had high perceived socioeconomic-related severity of hypertension, 1514 (62.5%) had a high perceived benefit of taking action, 1152 (47.5%) had low perceived barriers to taking action, and 1257 (51.9%) had high perceived self-efficacy of taking preventive measures (Table 3).

**Table 3:** Participants’ knowledge and beliefs about hypertension in rural areas of northwest Ethiopia, September-November 2020

Variable	Number of participants (%)	Hypertension, n (%)
Knowledge of common hypertensive symptoms		
Good	640 (26.4)	233 (30.7)
Moderate	280 (11.6)	96 (12.7)
Poor	1503 (62.0)	429 (56.6)
Knowledge of lifestyle risk factors and hypertensive prevention measures		
Good	1225 (50.6)	375 (49.5)
Moderate	496 (20.5)	155 (20.5)
Poor	702 (29.0)	228 (30.1)
Knowledge of biological and behavioral risk factors		
Good	208 (8.6)	78 (10.3)
Moderate	813 (33.6)	233 (30.7)
Poor	1402 (57.9)	447 (59.0)
Knowledge of the health consequences of hypertension		
Good	145 (6.0)	40 (5.3)
Moderate	1028 (42.4)	321 (42.4)
Poor	1,250 (51.6)	397 (52.4)
Overall knowledge of hypertension		
Good	496 (20.5)	175 (23.1)
Moderate	893 (36.9)	267 (35.2)
Low	1034 (42.7)	316 (41.7)
Perceived susceptibility		
High	957 (39.5)	344 (45.4)
Low	1466 (60.5)	414 (54.6)
Perceived health related severity		
High	1394 (57.5)	433 (57.1)
Low	1029 (42.5)	325 (42.9)
Perceived socioeconomic related severity		
High	1248 (51.5)	405 (53.4)
Low	1175 (48.5)	353 (46.6)
Perceived benefit of taking action		
High	1514 (62.5)	473 (62.4)
Low	909 (37.5)	285 (37.6)
Perceived barriers of taking action		
High	1271 (52.5)	396 (52.2)
Low	1152 (47.5)	362 (47.8)
Perceived self-efficacy		
High	1257 (51.9)	432 (57.0)
Low	1166 (48.1)	326 (43.0)

### Health service related characteristics of the participants

The health center was the nearest health facility to access healthcare services for most of participants (99.1%). The average distance and time to reach the nearest health facility were 6 km and 61 min, respectively. Of the participants, 1814 (74.9%) can reach the nearest health facility within 60 min and 2079 (85.8%) use foot to reach the nearest health facility. Only 654 (27.0%) of study participants have full community-based health insurance, 1198 (49.4%) have visited a health facility in the previous 12 months, with 1192 (99.5%) obtaining healthcare services for their own health (Table 4).

**Table 4:** Health services related characteristics of study participants in rural northwest Ethiopia, September-November 2020

Variables	Frequency	Percent
Nearest health facility		
Health center	2401	99.1
Private clinic	22	0.9
Travel distance to the nearest health facility		
≤ 5 km	1473	60.8
6–10 km	794	32.8
> 10 km	156	6.4
Travel time to the nearest health facility, in minutes		
≤ 60	1814	74.9
61–120	447	18.5
> 120 h	162	6.7
Thinking about distance to the nearest health facility		
Near	1518	62.7
Normal	481	19.9
Far	424	17.5
Thinking about travel time to the nearest health facility		
Short	1488	61.4
Normal	504	20.8
Long	431	17.8
Types of transport		
Public transportation	280	11.6
On foot	2079	85.8
Others*	64	2.6
Community based health insurance		
Adequately insured	654	27.0
Under insured	179	7.4
Uninsured	1590	65.6
Who covers the cost of the health care (n = 1769)		
From saving	364	20.6
Family	204	11.5
Fee waiver	37	2.1
Borrowing	220	12.4
Selling asset	944	53.4
Health facility visit during the last 12 months		
Yes	1198	49.4
No	1225	50.6
Health care utilization		
Yes	1192	99.5
No	6	0.5
Source of healthcare services (n = 1192)		
Health center	1078	90.4
Hospital	155	13.0
Private clinic	143	12. 0

*Horse or mule ride, traditional ambulance

### Proportion of undiagnosed hypertension

Of the total study participants, 758 (31.3%, 95% CI: 29.4–33.1%) had hypertension, with 637 (84.0%; 95% CI: 81.4–86.7%) having undiagnosed hypertension. Only 121(16.0%) of the participants with hypertension were aware of their diagnosis, and only 25 (3.3%) received treatment. The proportion of undiagnosed hypertension was higher among young adults and alcohol drinkers. But lower among participants having FHH, comorbidities, and higher Body Mass Index.

### Factors associated with undiagnosed hypertension

The multivariable binary logistic regression analysis in model 1 revealed that being young and having alcoholic drinks within the previous 12 months positively and significantly associated with undiagnosed hypertension. On the other hand, being overweight, having FHH, and comorbidities were negatively and significantly associated with undiagnosed hypertension. Hypertensive people aged 25–34 and 35–44 years were about six times (AOR = 6.03; 95% CI: 2.11, 17.29) and four times (AOR = 3.55; 95% CI: 1.66, 7.60) more likely to have undiagnosed hypertension than their older counterparts. Participants who had consumed alcohol in the previous 12 months were two times (AOR = 2.40; 95% CI: 1.37, 4.20) more likely to have undiagnosed hypertension as who had not. Overweight participants had a 59% (AOR = 0.41; 95% CI: 0.18, 0.98) lower risk of undiagnosed hypertension than participants with normal-weight participants. Participants with a FHH had a 68% (AOR = 0.32; 95% CI: 0.20, 0.53) lower proportion of undiagnosed hypertension than those without a FHH. Participants with comorbidities had a 72% (AOR = 0.28; 95% CI: 0.15, 0.54) lower proportion of undiagnosed hypertension than those without (Table 5).

**Table 5:** Bivariate and multivariable logistic regression analysis of factors associated with undiagnosed hypertension

Variables	Estimating path c				
	Undiagnosed hypertension		COR (95% CI)	^$^AOR(95% CI)	β
	Yes	No			
Age					
25–34	92 (93.9)	6 (6.1)	5.28 (2.18, 12.78)	6.03 (2.11, 17.29)	1.79**
35–44	137 (90.1)	15 (9.9)	3.15 (1.70, 5.84)	3.55 (1.66, 7.60)	1.27**
45–64	257 (84.3)	48 (15.7)	1.84 (1.19, 2.87)	2.03 (1.16, 3.55)	0.71*
≥65	151 (74.4)	52 (25.6)	1	1	
Alcoholic drink during the last 12 months					
Yes	581 (87.2)	85 (12.8)	4.39 (2.73, 7.08)	2.40 (1.37, 4.20)	0.88*
No	56 (39.1)	36 (60.9)	1	1	
Body Mass Index					
Underweight	187 (88.2)	25 (11.8)	1.45 (0.89, 2.33)	1.71 (0.99, 2.94)	0.54
Normal	429 (83.8)	83 (16.2)	1	1	
Overweight	21 (61.8)	13 (38.2)	0.31 (0.15, 0.65)	0.41 (0.18, 0.98)	-0.88*
Family history of hypertension					
Yes	83 (64.3)	46 (35.7)	0.24 (0.16, 0.38)	0.32 (0.20, 0.53)	-1.13**
No	554 (88.1)	75 (11.9)	1	1	
Comorbidities					
Yes	30 (50.8)	29 (49.2)	0.16(0.09, 0.27)	0.28 (0.15, 0.54)	-1.27**
No	607 (86.8)	92 (13.2)	1	1	

*P-value < 0.05 **P-value < 0.001 Hosmer and Lemeshow Test = 0.8958

^$^adjusted for sex, age, marital status, educational status, marital status, household income, alcohol consumption, physical activity, body mass index, family history of hypertension, comorbidities, health insurance, and travel time to nearest health facility

### Association between determinants and mediating variables

The regression analysis in model 2 revealed that participants who drank alcohol with in the previous 12 months were 52% (AOR = 0.48; 95% CI: 0.28, 0.80) less likely to have hypertension health information than non-drinkers. Participants with a FHH (AOR = 2.45; 95% CI: 1.59, 3.79) and comorbidities (AOR = 2.48; 95% CI: 1.33, 4.62) were twice as likely as their counterparts to have hypertension health information. Participants with a FHH were twice (AOR = 2.01; 95% CI: 1.34, 3.03) more likely to have good knowledge of common hypertensive symptoms than those without. Participants who had access to hypertension health information were five times (AOR = 4.97; 95% CI: 3.48, 7.11) more likely to have good knowledge of common hypertensive symptoms than those who did not. Young participants were 61% (AOR = 0.39; 95% CI: 0.20, 0.75) less likely than the elderly to have a high perceived susceptibility to hypertension. Alcohol drinkers were also 48% (AOR = 0.52; 95% CI: 0.30, 0.90) less likely to visit a health facility than non-drinkers, while participants with comorbidities were two times (AOR = 2.07; 95% CI: 1.04, 4.15) more likely to visit a health facility than non-comorbid participants (Supplementary File).

### Associations between mediating variables and undiagnosed hypertension

In model 3, adequate hypertension health information, knowledge of common hypertensive symptoms, perceived susceptibility to hypertensive disease, and health facility visit were significantly associated with undiagnosed hypertension while controlling for the independent variables and covariates. Patients with hypertension who had adequate hypertension health information had a 68% (AOR = 0.32; 95% CI: 0.19, 0.55) lower risk of undiagnosed hypertension compared to participants who did not have any hypertension health information. Patients with hypertension who had a good knowledge of common hypertensive symptoms had a 72% (AOR = 0.28; 95% CI: 0.14, 0.53) lower proportion of undiagnosed hypertension than those who did not. Participants with a high perceived susceptibility to hypertensive disease have a 42% (AOR = 0.58; 95% CI: 0.35, 0.97) lower proportion of undiagnosed hypertension than those who did not. Participants who visited a health facility have a 60% (AOR = 0.40; 95% CI: 0.24, 0.69) lower proportion of undiagnosed hypertension than those did not (Supplementary File).

### Mediation analysis

**Fig. 1:**
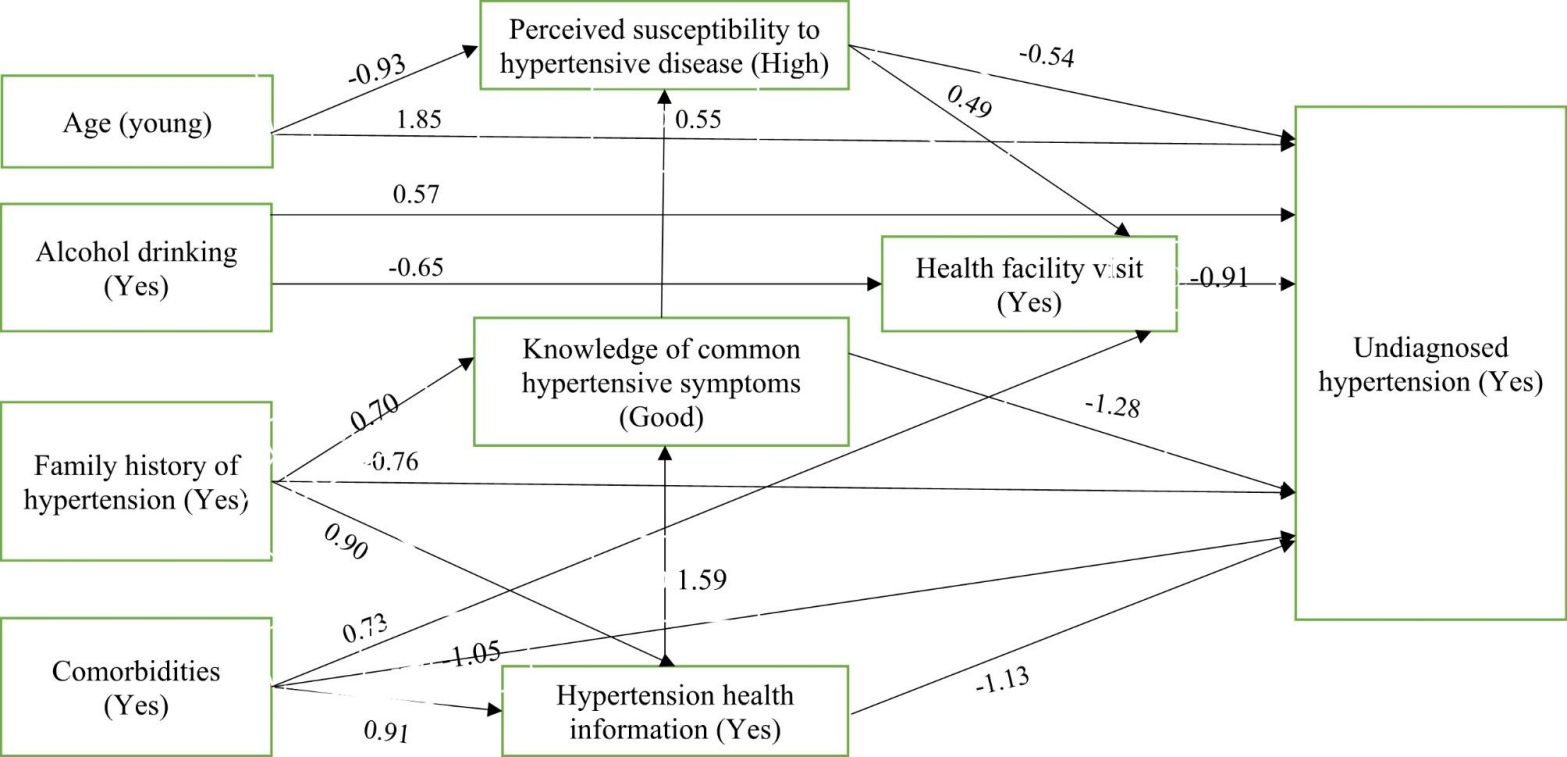
Path diagram showing mediating variables mediates the association between undiagnosed hypertension and its determinants. The arrow represents the beta coefficients of the relationship between variables

The study found that the association between age and undiagnosed hypertension was mediated in part by perceived susceptibility to hypertensive disease, as well as its association with poor health facility visits. Poor perceived susceptibility to hypertensive disease accounted for 18.1% of the relationship between young age and undiagnosed hypertension. Poor perceived susceptibility to hypertensive disease and poor health facility visit in combination explained 33.3% of the association between young age and undiagnosed hypertension. Poor health facility visit mediated 14.2% of the relationship between alcohol consumption and undiagnosed hypertension and 12.3% of the relationship between comorbidities and undiagnosed hypertension. After controlling for mediators, the effect of alcohol drinking within the previous 12 months on undiagnosed hypertension was no longer significant (β=0.57, p = 0.09), indicating that this relationship has no direct effect. Hypertension health information mediated 64.1% of the total effect of FHH on lower proportion of undiagnosed hypertension, both independently and through the other 3 mediators (knowledge of common hypertensive symptoms, perceived susceptibility to hypertensive disease, and visits to a health facility). Knowledge of common hypertensive symptoms mediated 22.4% of the total effect of FHH on lower proportion of undiagnosed hypertension, both independently and through the other 2 mediators (perceived susceptibility to hypertensive disease and visits to a health facility). Hypertension health information also mediated 68.2% of the total effect of having comorbidities on lower proportion of undiagnosed hypertension through its association with undiagnosed hypertension and through the other 3 mediators (knowledge of common hypertensive symptoms, perceived susceptibility to hypertensive disease, and visits to a health facility) (Fig. 1).

## Discussion

In this study, we found a high prevalence of hypertension (31.3%) with a very low awareness rate (16.0%). This reveal that unable to meet Ethiopia’s target of improving the proportion of hypertensive adults diagnosed and aware of their status to 50% by 2022 [2]. The study found that factors such as young age, drinking alcohol, being overweight, having FHH and comorbidities were all independently associated with undiagnosed hypertension.

In this study, nearly 9 out of 10 of patients with hypertension remained undiagnosed, implying that they were hiding in plain sight. This finding was supported by studies conducted in Kenya (84.4%) [41], rural Nigeria (81.5%) [42], and rural areas of Southern China (82.4%) [43]. This could be because the same age group and sampling strategy were used. However, the proportion of undiagnosed hypertension was higher than in the 2018 Ethiopian non communicable disease and injuries commission report (60%) [1], and in studies conducted in Dabat district northwest Ethiopia (59.7%) [4], Amhara regional state cities (46.3%) [44], Axum town (57%) [45], and Nekemte town (47.3%) [46]. The high proportion of undiagnosed hypertension in this study could be explained by the fact that it was only conducted on rural adults, who are up to 2 times more likely than urban adults to have undiagnosed hypertension [47–49]. It was also higher than studies conducted in African rural communities in Tanzania (51.7% and 65.7%) [50, 51], Uganda (61.9%, 70.3%, and 79.0%) [52–54], Cameroon (36.6% and 62.8%) [55, 56], Ghana (54.4%) [57], Northern Senegal (56.7%) [58], Benin (58.0%) [59], and South Africa (35.6%) [60]. This might be because the lower cut-off point used to classify hypertension in this study, which may have increased both the prevalence of hypertension and undiagnosed hypertension [61]. However, the proportion of undiagnosed hypertension in this study was lower than the studies conducted in rural parts of northwest Tanzania (93%) [49] and findings from the Uganda national noncommunicable disease risk factor survey (94%) [62]. This could be because the majority of participants, 75.1% in Tanzania and 66% in Uganda, were between the ages of 15 and 44 years and18 and 40 years, respectively, and being young was associated with undiagnosed hypertension.

The age of the participants was found to be strongly associated with undiagnosed hypertension. Undiagnosed hypertension was about six and four times more common in hypertensive patients aged 25–34 and 35–44 years, respectively, than those aged 65 years and older. The finding were consistent with previous research from Kenya [41], Tanzania [50], Egypt [63], Cameroon [64], Gambia [65], South Africa [60], Peru [66], Bangladesh [67], Kurdish adults [68], and Iran [69, 70]. This could be due to the poor belief that they have a low risk of developing hypertension [71], which makes them less likely to visit a health facility and have their BP measured. The association between age and undiagnosed hypertension was partially mediated by perceived susceptibility to hypertensive disease, which explained 18.1% of the relationship. Perceived susceptibility to hypertensive disease with its association with poor health facility visit also explained 15.2% of the relationship. Both mediators together explained 33.3% of the association. Other studies have also found that people with a low perceived susceptibility to hypertensive disease visit the health facility less frequently and have lower rates of screening and diagnosis [72]. The remaining effect of age on undiagnosed hypertension could be attributed to the fact that young adults are less likely than the elderly to have comorbidities, seek medical advice, be informed about their BP, and be diagnosed with hypertension at younger age.

Despite the fact that people who drink excessive amounts of alcohol are more likely to develop hypertension [7, 73, 74] and should have their BP checked, this study found that participants who consumed alcohol within the previous 12 months were 2.4 times more likely to have undiagnosed hypertension compared with the counterparts. This finding was consistent with those of studies in rural Tanzania [50] and China [75, 76] where alcohol drinkers had higher rates of undiagnosed hypertension than non-drinkers. Our findings revealed that those who drank alcohol within the previous 12 months were 48% less likely to visit a health facility, and those who did not visit a health facility in the previous year were 2.5 times more likely to have undiagnosed hypertension. As a result, inadequate healthcare visits account for about 14.2% of the overall effect of alcohol consumption on the increased risk of undiagnosed hypertension. Another finding also revealed alcohol drinkers used less health care [77], implying that patients with hypertension who did not visit a health facility resulted more undiagnosed hypertension [78, 79].

Overweight participants had better hypertension awareness compared to those of normal weight. Similar empirical evidences generated from Kenya [80], Tanzania [81], Egypt [63], Gambia [65], Ghana [82], Peru [66], Bangladesh [67], Iran [70, 83], Nepal [84], and Northeast China [76] found that overweight or obese participants were more likely to be aware of their hypertension status than normal-weight participants. This could be because being overweight is a known risk factor for hypertension and other comorbidities and people who are overweight are more likely to be screened and have their BP measured [85].

Participants who had a FHH were 68% less likely to have undiagnosed hypertension than those who did not. This finding was consistent with studies from Cameroon [64], Ghana [82], Iran [68], Jordan [86], and Southern China [43]. This could be because participants with a FHH may have hypertension health information and gain knowledge from their relatives’ experiences [87, 88], and improving knowledge has been shown to improve health seeking behavior and hypertension diagnosis by improving perceptions of individuals’ susceptibility to hypertensive disease [89, 90]. Our findings also revealed that having hypertension health information, a good knowledge of common hypertensive symptoms, a high perceived susceptibility to hypertensive disease, and adequate health facility visit explained the association between having a FHH and lower proportion of undiagnosed hypertension. Having hypertension health information explained 64.1% of the total effect of FHH on lower proportion of undiagnosed hypertension in this study. Having a good knowledge of common hypertensive symptoms also explained 22.4% of the total effect of FHH on lower proportion of undiagnosed hypertension via its association with high perceived susceptibility to hypertensive disease and visiting a health facility. This finding suggests that providing hypertension health information is critical for increasing knowledge and perceived susceptibility to hypertensive disease, encouraging people to seek medical advice, and resulting in hypertension being diagnosed earlier.

Patients with comorbidities have a higher rate of hypertension detection and a lower proportions of undiagnosed hypertension [68, 79, 91]. In this study, patients with comorbidities had a 72% lower risk of undiagnosed hypertension than patients without comorbidities. This was consistent with findings from studies in South Africa [60], Ghana [82], Lebanon [92], and Iran [70] in which participants with prior CVD comorbidities had a lower rate of undiagnosed hypertension than those without CVD comorbidities. Studies conducted in Ibadan Nigeria [93] and Iran [94] also showed that patients with history of diabetes were less likely to have undiagnosed hypertension. Our findings revealed that participants with comorbidities were twice as likely to have hypertension health information. Hypertension health information is a critical component in the early detection and treatment of hypertension. Having hypertension health information explained 68.2% of total effect of comorbidities on lower proportion of undiagnosed hypertension in combination with good common hypertensive symptoms, perceived susceptibility to hypertensive disease and visiting health facility. Participants with comorbidities were also twice as likely to visit a health facility, and participants who had visited a health facility in the previous one year were 60% less likely than those who had not to have undiagnosed hypertension. As a result, adequate health facility visitation mediated 12.3% of the total effect of comorbidities on the reduction of undiagnosed hypertension risk. This findings were consistent with previous studies that found that visiting a health facility encourages people to seek medical advice and undergo hypertension screening, resulting in hypertension being diagnosed earlier [95, 96]. This finding was also consistent with findings from studies conducted in Ghana [57], Senegal [97], India [78], and China [79] in which seeking outpatient services in the previous 4 weeks was associated with increased hypertension awareness.

## Strengths and limitations

The study has the following strengths: first, it is the first to use mediation analysis to investigate the potential mechanisms of factors affecting undiagnosed hypertension. Second, it employs of a large representative sample. Third, it uses a valid and reliable tool to assess health measures. However, due to the study’s cross-sectional design, we are unable to assess the temporal relationship between the exposure, mediator, and outcome variables.

## Conclusion

In this study, undiagnosed hypertension remains very high in rural northwest Ethiopia. Being young, drinking alcohol within the previous 12 months, being overweight, having FHH, and having comorbidities were found to be independently associated with undiagnosed hypertension. Hypertension health information, knowledge of common hypertensive symptoms, perceived susceptibility to hypertensive disease, and health facility visit mediated predictors of undiagnosed hypertension. Public health interventions aimed at them, such as providing adequate hypertension health information, particularly to young adults and drinkers, may improve hypertensive disease knowledge, perceived susceptibility to hypertensive disease, and reduce the burden of undiagnosed hypertension.

## Electronic supplementary material

Below is the link to the electronic supplementary material.

Supplementary Material 1

## Data Availability

This manuscript contains all of the data generated or analyzed during the study. However, the de-identified datasets used in the reported study are available upon reasonable request from the corresponding author.

## Abbreviations

AOR: Adjusted Odd Ratio.
BP: Blood Pressure.
CI: Confidence Interval.
CVD: Cardiovascular Diseases.
FHH: Family History of Hypertension.
IRB: Institutional Review Board.
WHO: World Health Organization.
