# Supplementary material for: Undiagnosed hypertension and its determinants among hypertensive patients in rural districts of northwest Ethiopia: a mediation analysis
Source: BMC Health Serv Res. 2023 Mar 7;23:222. doi: 10.1186/s12913-023-09212-1 (PMC9990316; doi:10.1186/s12913-023-09212-1)
Supplement: Supplementary file 1 — Supplementary Material 1 [file 12913_2023_9212_MOESM1_ESM.docx]

Table 6 Multivariable logistic regression analysis of factors affecting undiagnosed hypertension adjusted for covariates and mediators

| **Variables** | **Estimating path a** | | | | | | | | **Estimating path b and c’** | |  |
| --- | --- | --- | --- | --- | --- | --- | --- | --- | --- | --- | --- |
|  | **Hypertension Health information** | | **Knowledge of hypertension symptoms** | | **Perceived susceptibility to hypertension** | | **Health facility visit** | | **Undiagnosed hypertension** | | **Types of mediation** |
|  | **AOR(95% CI)** | **ꞵ** | **AOR(95% CI)** | **ꞵ** | **AOR(95% CI)** | **ꞵ** | **AOR(95% CI)** | **ꞵ** | ^$^**AOR(95% CI)** | **ꞵ** |  |
| Age |  |  |  |  |  |  |  |  |  |  |  |
| 25-34 | 0.83 (0.41, 1.69) | -0.53 | 1.89 (0.99, 3.40) | 0.64 | 0.39 (0.20, 0.75) | -0.93** | 0.86 (0.45, 1.64) | -0.16 | 6.33 (2.04, 19.69) | 1.85** | Partial |
| 35-44 | 1.09 (0.59, 1.98) | 0.08 | 1.23 (0.74, 2.04) | 0.21 | 0.95 (0.57, 1.61) | -0.05 | 1.10 (0.64, 1.90) | 0.10 | 4.39 (1.90, 10.12) | 1.48** |  |
| 45-64 | 1.08 (0.65, 1.78) | 0.07 | 1.02 (0.67, 1.57) | 0.02 | 0.78 (0.50, 1.19) | -0.25 | 0.94 (0.60, 1.48) | -0.06 | 2.13 (1.15, 3.92) | 0.76* |  |
| ≥65 | 1 |  | 1 |  | 1 |  | 1 |  | 1 |  |  |
| Alcoholic drink during the last 12 months |  |  |  |  |  |  |  |  |  |  |  |
| Yes | 0.48 (0.28, 0.80) | -0.74* | 0.62 (0.38, 1.01) | -0.48 | 1.09 (0.66, 1.81) | 0.09 | 0.52 (0.30, 0.90) | -0.65* | 1.77 (0.92, 3.40) | 0.57 | Partial |
| No | 1 |  | 1 |  | 1 |  | 1 |  | 1 |  |  |
| Body Mass Index |  |  |  |  |  |  |  |  |  |  |  |
| Underweight | 1.09 (0.73, 1.64) | 0.09 | 0.92 (0.64, 1.30) |  | 1.03 (0.72, 1.46) | 0.03 | 1.27 (0.89, 1.82) | 0.24 | 1.73 (0.96, 3.11) | 0.55 |  |
| Normal | 1 |  | 1 |  | 1 |  | 1 |  | 1 |  |  |
| Overweight | 0.95 (0.40, 2.27) | -0.05 | 1.11 (0.52, 2.36) |  | 0.95 (0.45, 2.02) | -0.05 | 0.61 (0.28, 1.34) | -0.49 | 0.30 (0.11, 0.83) | -1.19* | No |
| Family history of hypertension |  |  |  |  |  |  |  |  |  |  |  |
| Yes | 2.45 (1.59, 3.79) | 0.90** | 2.01 (1.34, 3.03) | 0.70** | 1.27 (0.83, 1.94) | 0.24 | 1.53 (0.97, 2.42) | 0.43 | 0.47 (0.27, 0.81) | -0.76** | Partial |
| No | 1 |  | 1 |  | 1 |  | 1 |  | 1 |  |  |
| Comorbidities |  |  |  |  |  |  |  |  |  |  |  |
| Yes | 2.48 (1.33, 4.62) | 0.91** | 1.02 (0.57, 1.86) | 0.02 | 1.75 (0.94, 3.26) | 0.56 | 2.07 (1.04, 4.15) | 0.73* | 0.35 (0.17, 0.72) | -1.05** | Partial |
| No | 1 |  | 1 |  | 1 |  | 1 |  | 1 |  |  |
| Hypertension health information |  |  |  |  |  |  |  |  |  |  |  |
| Yes |  |  | 4.97 (3.48, 7.11) | 1.59** | 1.23 (0.82, 1.84) | 0.21 | 1.04 (0.68, 1.58) | 0.04 | 0.32 (0.19, 0.55) | -1.13** |  |
| No |  |  | 1 |  | 1 |  | 1 |  | 1 |  |  |
| Knowledge of common hypertension symptoms |  |  |  |  |  |  |  |  |  |  |  |
| Good |  |  |  |  | 1.74 (1.13, 2.67) | 0.55* | 1.06 (0.68, 1.64) | 0.06 | 0.28 (0.14, 0.53) | -1.28** |  |
| Moderate |  |  |  |  | 0.95 (0.57, 1.59) | -0.05 | 1.40 (0.83, 2.45) | 0.33 | 0.28 (0.13, 0.58) | -1.28** |  |
| Poor |  |  |  |  | 1 |  | 1 |  | 1 |  |  |
| Perceived susceptibility to hypertension |  |  |  |  |  |  |  |  |  |  |  |
| High |  |  |  |  |  |  | 1.64 (1.198 2.28) | 0.49** | 0.58 (0.35, 0.97) | -0.54* |  |
| Low |  |  |  |  |  |  | 1 |  | 1 |  |  |
| Health facility visit |  |  |  |  |  |  |  |  |  |  |  |
| Yes |  |  |  |  |  |  |  |  | 0.40 (0.24, 0.69) | -0.91** |  |
| No |  |  |  |  |  |  |  |  | 1 |  |  |

AOR=Adjusted odds ratio, CI=Confidence interval, ꞵ=beta coefficients, *P-value<0.05, and **P-value<0.001

^$^adjusted for sex, age, marital status, educational status, marital status, household income, alcohol consumption, physical activity, Body Mass Index , FHH, comorbidities, hypertension health information, health insurance, and travel time to nearest health facility, knowledge of lifestyle risk factors, knowledge of consequences of hypertension, perceived health related severity of hypertension, and perceived benefit of taking action
